# Analysis of current status and influencing factors of health promotion behaviors among patients after PCI: based on health ecology theory

**DOI:** 10.3389/fpubh.2025.1698957

**Published:** 2025-12-11

**Authors:** Yuxin Li, Tianxia Zhao, Ping Dai, Yanhong Wen, Yuting Fan, Jijun Wu, Lin He

**Affiliations:** 1 Department of Nursing, Deyang People’s Hospital, Deyang, Sichuan, China; 2 Department of Respiratory and Critical Care, Deyang People’s Hospital, Deyang, Sichuan, China; 3 Department of Cardiology, Deyang People’s Hospital, Deyang, Sichuan, China

**Keywords:** percutaneous coronary intervention, health promotion behaviors, health ecology theory, influencing factors analysis, nursing

## Abstract

**Background:**

Coronary heart disease (CHD) is one of the most common cardiovascular diseases, with its prevalence and mortality rates increasing annually. Percutaneous coronary intervention (PCI) serves as a crucial revascularisation method for CHD, effectively alleviating myocardial ischemia and improving patient outcomes. However, patients undergoing PCI often experience adverse events such as restenosis due to unhealthy lifestyles or behaviors. Therefore, conducting a comprehensive, multifaceted exploration of the factors influencing health promotion behaviors in post-PCI patients is crucial for enhancing these behaviors and preventing adverse cardiovascular events.

**Objective:**

Based on health ecology theory, this study aims to understand the current status of health promotion behaviors among post-PCI patients, analyze their influencing factors, and provide evidence for developing targeted interventions to improve these behaviors.

**Methods:**

This cross-sectional study employed a convenience sampling approach. Between March and October 2024, 436 patients with PCI from the Cardiovascular Department of a Grade A tertiary general hospital in Sichuan Province, China, were enrolled. The study employed a general information questionnaire, the health promotion lifestyle scale, the health literacy scale, the psychological resilience scale, and the perceived social support scale for data collection. Univariate analysis, correlation analysis, and multiple stepwise linear regression were used to explore the factors influencing health promotion behaviors among these patients.

**Results:**

The mean health promotion behavior score among PCI patients was (84.55 ± 11.86). Correlation analysis revealed positive associations between health promotion behaviors and health literacy, psychological resilience, and perceived social support (*r* = 0.747–0.809, *p* < 0.01). Multivariate stepwise linear regression analysis revealed that age, occupational status, self-rated sleep quality, whether the patient or family members have worked in healthcare, receipt of chronic disease knowledge guidance, health literacy, psychological resilience, and perceived social support were significant factors influencing health promotion behaviors (*p* < 0.05), explaining 77.6% of the total variance.

**Conclusion:**

Health promotion behaviors among PCI patients post-surgery are at a moderate level. Influencing factors are multi-level and multidimensional, suggesting comprehensive interventions targeting individuals, families, and society are necessary to enhance health promotion behaviors.

## 1 Introduction

Cardiovascular diseases have become a significant global public health issue, severely impacting patients’ physical and mental health as well as their quality of life, while imposing a heavy economic burden on families, society, and nations (1). Coronary heart disease (CHD) is one of the most common cardiovascular conditions, with its prevalence and mortality rates rising annually. Survey data indicate that as of 2019, approximately 197 million people worldwide suffered from CHD, with the prevalence projected to increase by 18.0% by 2030 (2). In China, the current number of CHD patients stands at 11.39 million, and it is estimated that by 2035, over 3.4 million people in the country will die from CHD (3). Percutaneous coronary intervention (PCI) refers to the use of cardiac catheterisation techniques to clear narrowed or even blocked coronary artery lumens, thereby improving myocardial blood flow perfusion (4). It is currently recognized in the medical field as an effective treatment for CHD. Globally, approximately 5 million CHD patients undergo PCI annually. In China, PCI procedures have increased from 228,000 in 2009 to 1.421 million in 2022, accounting for 23.2% of all CHD hospitalizations (5). However, PCI does not eliminate the risk factors for coronary heart disease. Post-PCI patients often experience adverse events such as restenosis due to unhealthy lifestyles or behaviors (6). Reports indicate an in-stent restenosis rate of 2 to 10% among patients undergoing PCI (7). Furthermore, multiple studies confirm that the occurrence of adverse cardiovascular events post-PCI is closely linked to health promotion behaviors (8, 9). Therefore, conducting in-depth, multifaceted research on the factors influencing health promotion behaviors in post-PCI patients is crucial for enhancing these behaviors and preventing adverse cardiovascular events.

Health promotion behaviors refer to a series of actions individuals undertake to enhance or maintain their health. These encompass six key areas: nutrition, physical activity, health responsibility, interpersonal relationships, mental growth, and stress management (10). Such behaviors can prevent disease and sustain or improve an individual’s health status to a certain extent. For patients who have undergone PCI, actively practicing health-promoting behaviors is crucial. Relevant studies indicate that adhering to guideline-recommended lifestyle modifications (such as the Mediterranean diet and regular exercise) and secondary prevention medications can reduce the risk of recurrent major adverse cardiovascular events by up to 80%, while significantly improving patients’ quality of life and long-term survival rates (11). However, multiple global studies consistently reveal that the current state of health promotion behaviors among PCI patients is generally concerning, posing a major challenge in contemporary cardiovascular disease management. For instance, a survey of Chinese PCI patients found that only 24.2% demonstrated overall good health behaviors, with particularly poor adherence to physical activity and health responsibility (12). European research indicates that up to 38% of PCI patients lack motivation to alter personal lifestyle habits, with only approximately 27% acknowledging the need for behavioral changes to manage cardiovascular disease (13). This “knowledge–behavior gap”—where patients understand health information but struggle to translate it into sustained action—significantly undermines the long-term efficacy of PCI procedures. Furthermore, related studies indicate that most PCI patients exhibit unhealthy lifestyles and behaviors, including insufficient physical activity, poor dietary patterns, and non-compliant medication use (14). These detrimental health-promoting behaviors are associated with multiple factors, including personal characteristics (such as gender, age, and educational attainment) and disease-related factors (such as the number of chronic conditions and disease duration) (15, 16). More critically, poor health-promoting behaviors among PCI patients lead to direct negative clinical outcomes. Studies confirm that behaviors such as discontinuing medication without medical advice, smoking, and lack of exercise are independent risk factors for restenosis and readmission following PCI (17, 18). These detrimental behaviors not only compromise patients’ physical and mental health and quality of life but also significantly increase the risk of revascularization and all-cause mortality, imposing a heavy economic burden on families and society (19, 20). Therefore, thoroughly investigating the multi-level factors influencing health promotion behaviors among PCI patients and developing targeted intervention strategies based on relevant theories holds immense clinical necessity and public health significance.

Current research on factors influencing health promotion behaviors among patients after PCI procedures primarily focuses on the impact of individual factors on health behaviors, lacking a systematic analysis of the combined effects of multi-level factors. Health ecology theory, proposed by McLeroy et al. (21) as a derivative of ecological theory, emphasizes that the combined influence of multi-level, multidimensional factors shapes individual health behaviors. These include the personal characteristics level, psychological and behavioral characteristics level, interpersonal network level, living and working conditions level, and policy environment level. This theory applies ecological thinking and theoretical frameworks to population health research, emphasizing that individual health is shaped by the interplay, interdependence, and mutual constraints of personal factors, healthcare services, and social policy environments. It is now widely applied in health promotion studies for chronic diseases and special populations (22, 23). Therefore, this study, grounded in health ecology theory, explores the determinants of health promotion behaviors among PCI patients from multiple perspectives. It aims to provide evidence for developing targeted interventions to enhance health promotion behaviors in this population.

## 2 Methods

### 2.1 Study design

This study is a cross-sectional study.

### 2.2 Participants

Using convenience sampling, patients hospitalized in the Cardiovascular Department of a Grade A tertiary general hospital in Sichuan Province, China, who underwent PCI between March and October 2024 were selected as study subjects. Inclusion criteria: ① Patients meeting diagnostic and treatment criteria for coronary heart disease, fulfilling indications for PCI (24), having completed the PCI procedure, and exhibiting stable vital signs; ② Patients aged ≥18 years; ③ Patients who are fully conscious possess normal cognitive function and are capable of verbal or written communication. ④ Patients who provided informed consent and voluntarily participated in the study. Exclusion criteria: ① Patients with severe concomitant systemic diseases, such as liver, brain, or kidney dysfunction, tumor recurrence/metastasis, or severe infectious diseases. ② Patients unable to complete this study survey for any reason (e.g., severe audiovisual impairment, aphasia, altered mental status, etc.).

According to the sample size calculation formula (25): *n = (u_1 -α/2_ σ/δ)*^2^, with a significance level α = 0.05, then *u_1 -α/2_* = 1.96. Based on preliminary survey results, the standard deviation (*α*) for health promotion behaviors among PCI postoperative patients is 0.32. The permissible error *δ* is set at 10% of the standard deviation α, i.e., δ = 0.032. Considering a 10% non-response rate, the planned sample size was 428 cases. Ultimately, 436 cases were included, meeting the requirements.

### 2.3 Variable selection

This study constructs an analytical framework based on health ecology theory. The selection of research variables at each level is based on the following principles: First, by reviewing literature on health promotion behaviors among patients after PCI (9, 12, 13, 16), factors that repeatedly emerge across levels and are proven to have a significant influence were preliminarily identified. Second, these factors were refined and integrated considering the specific context of this study and the availability of data. Therefore, the influencing factors of health promotion behaviors among patients after PCI in this study are constructed from the following aspects:

(1) Individual Traits Layer: Gender, age, BMI, number of chronic diseases, disease duration, family history of cardiovascular disease, previous hospitalizations for coronary heart disease, number of interventional treatments, number of stent implants, health literacy; (2) Psychological and Behavioral Characteristics Layer: smoking situation, drinking situation, self-reported sleep quality, psychological resilience; (3) Interpersonal Network Layer: marital status, number of children, receipt of chronic disease knowledge guidance, perceived social support; (4) Work and Living Conditions Layer: education level, average monthly family income, occupational status, place of residence, housing conditions, whether the patient or family member has worked in healthcare; (5) Policy Environment Layer: medical payment methods.

### 2.4 Measures

#### 2.4.1 General information questionnaire

Designed by the researcher through literature review and clinical practice, based on thorough discussion with the research team. Content primarily includes: gender, age, BMI, number of chronic diseases, disease duration, family history of cardiovascular disease, previous hospitalizations for coronary heart disease, number of interventional treatments, number of stent implants, smoking situation, drinking situation, self-reported sleep quality, marital status, number of children, receipt of chronic disease knowledge guidance, education level, average monthly family income, occupational status, place of residence, housing conditions, whether the patient or family members have worked in healthcare, medical payment methods.

#### 2.4.2 Health promotion lifestyle scale

The Health Promotion Lifestyle Scale was developed by American scholars Walker et al. (26) and subsequently adapted for use in China by Chinese scholars Cao et al. (27). It is primarily used to assess individuals’ health-promoting behaviors. The scale comprises 40 items across six dimensions: nutrition (6 items), physical activity (8 items), health responsibility (11 items), interpersonal relationships (5 items), spiritual growth (5 items), and stress management (5 items). It employs a 4-point Likert scale ranging from “Never” (1 point) to “Always” (4 points), yielding a total score between 40 and 160 points. Higher scores indicate stronger health-promoting behaviors. The scale’s Cronbach’s *α* coefficient is 0.930, which is consistent with the value of 0.941 reported in this study.

#### 2.4.3 Health literacy scale

The health literacy scale was developed by Jordan et al. (28) and adapted into Chinese by Chinese scholar Sun (29). This scale comprises four dimensions: information acquisition ability (9 items), communication and interaction ability (9 items), willingness to improve health (4 items), and willingness to seek financial support (2 items), totaling 24 items. The scale employs a 5-point Likert scale, scoring from “very difficult” to “not difficult at all” as 1 to 5 points, respectively. The total score ranges from 24 to 120 points, with higher scores indicating greater health literacy. The Cronbach’s *α* coefficient for this scale is 0.890. In this study, the Cronbach’s α coefficient for the scale was 0.944.

#### 2.4.4 Psychological resilience scale

The psychological resilience scale was developed by Campbell-Sills and Stein (30) and adapted into Chinese by Wang et al. (31). This unidimensional scale comprises 10 items. It employs a 5-point Likert scale ranging from “Never” to “Always,” scored from 0 to 4 points, respectively. The total score ranges from 0 to 40 points, with higher scores indicating greater psychological resilience. The scale’s Cronbach’s *α* coefficient is 0.910. In this study, the Cronbach’s α coefficient for this scale was 0.863.

#### 2.4.5 Perceived social support scale

The perceived social support scale was developed by Blumenthal et al. (32) and adapted into Chinese by Huang et al. (33). This scale comprises three dimensions: family support (4 items), friend support (4 items), and other support (4 items), totaling 12 items. The scale employs a 7-point Likert scale, scoring items from “strongly disagree” to “strongly agree” with values ranging from 1 to 7. The total score ranges from 12 to 84 points, with higher scores indicating greater perceived levels of social support. The Cronbach’s *α* coefficient for this scale is 0.992. In this study, the Cronbach’s α coefficient for the scale was 0.898.

### 2.5 Data collection

This study employed paper-based questionnaires for data collection. Before the formal survey, a pre-survey was conducted with subjects who met the inclusion criteria to assess the suitability of the questionnaire. Based on feedback from these subjects, the questionnaire content was revised and refined. During the formal survey, standardized instructions were provided to participants, detailing the study’s purpose, significance, questionnaire completion methods, and precautions. After obtaining informed consent and signed consent forms, paper questionnaires were distributed and completed in patient wards. For participants with lower educational attainment or those unable to complete the questionnaire independently, researchers assisted by reading each question aloud to them. Researchers compiled disease-specific data by reviewing electronic medical records or by inquiring with patients. Throughout the questionnaire completion process, researchers avoided prompting or interfering with patients. Upon completion, researchers collected and verified each questionnaire on-site. Any omissions were addressed immediately by requesting participants to supplement the information. All data were entered and cross-checked by two individuals to ensure accuracy and consistency. A total of 450 questionnaires were collected for this study. After excluding 14 questionnaires with logical errors or consistent patterns of responses, 436 valid questionnaires were ultimately recovered, yielding a valid response rate of 96.9%.

### 2.6 Statistical methods

Data analysis was performed using SPSS 26.0 software. Categorical data were described using frequency, percentage, or proportion. For continuous data following a normal distribution, mean ± standard deviation was used for description, and between-group comparisons employed independent samples *t*-tests or one-way ANOVA. For continuous data that did not follow a normal distribution, the median (interquartile range) was used for description, and between-group comparisons utilized non-parametric tests. For correlation analysis, Spearman’s rank correlation was used for non-normally distributed or ordinal data. Pearson’s correlation analysis was applied to normally distributed continuous variables to investigate the relationships between health promotion behaviors, health literacy, psychological resilience, and perceived social support. Multivariate stepwise linear regression analysis was employed to identify factors influencing health promotion behaviors among PCI patients. *p* < 0.05 was considered statistically significant.

### 2.7 Ethical considerations

This study adheres to the Declaration of Helsinki and has been approved by the Ethics Committee of Deyang People’s Hospital (Approval No. 2024–04-018-K01). All research subjects provided informed consent and voluntarily participated in this study.

## 3 Results

### 3.1 General information on patients after PCI

Among the 436 patients undergoing PCI in this study, males accounted for 58.9% and females for 41.1%. The majority were aged 70 years or older (39.2%), and most were married (79.8%). Additional demographic characteristics are summarized in Table 1.

**Table 1:** Univariate analysis of health promotion behaviors among patients after PCI (*n* = 436).

Items	*N*(%)	Mean ± SD	*t/F*	*P*
Gender			−0.781	0.435
Male	257(58.9)	84.18 ± 11.64		
Female	179(41.1)	85.08 ± 12.17		
Age (years)			35.434	<0.001
<60	137(31.4)	89.83 ± 10.23		
60 ~ <70	128(29.4)	86.23 ± 9.38		
≥70	171(39.2)	79.06 ± 12.45		
BMI (kg/m^2^)			3.821	0.010
<18.5	16(3.7)	76.69 ± 11.82		
18.5 ~ 23.9	209(47.9)	83.78 ± 11.94		
24.0 ~ 27.9	171(39.2)	86.20 ± 11.86		
≥28	40(9.2)	84.68 ± 9.23		
Marital status			8.658	<0.001
Married	348(79.8)	86.83 ± 10.85		
Single/Divorced/Widowed	88(20.2)	75.51 ± 11.38		
Number of children			2.338	0.073
0	2(0.5)	95.00 ± 8.49		
1	258(59.2)	85.37 ± 11.86		
2 ~ 3	165(37.8)	83.55 ± 11.81		
≥4	11(2.5)	78.36 ± 10.26		
Education level			54.660	<0.001
Elementary and below	231(53.0)	79.89 ± 10.60		
Junior	113(25.9)	87.24 ± 10.47		
High school and above	92(21.1)	92.95 ± 10.40		
Occupational status			4.699	<0.001
Retirement	336(77.1)	83.27 ± 12.26		
Employed	100(22.9)	88.83 ± 9.80		
Place of residence			9.349	<0.001
Towns	221(50.7)	89.33 ± 11.35		
Countryside	215(49.3)	79.63 ± 10.26		
Medical payment methods			42.146	<0.001
Urban medical insurance	189(43.3)	90.05 ± 11.18		
Rural medical insurance	152(34.9)	80.94 ± 9.76		
Self-funded	95(21.8)	79.55 ± 11.79		
Average monthly family income (yuan)			103.551	<0.001
<3,000	57(13.1)	75.72 ± 11.49		
3,000 ~ <5,000	260(59.6)	82.00 ± 10.45		
≥5,000	119(27.3)	94.35 ± 8.17		
Housing conditions			31.395	<0.001
Living alone	22(5.1)	83.00 ± 13.55		
Living with children	63(14.4)	74.62 ± 10.88		
Living with spouse	206(47.2)	83.87 ± 10.87		
Living with children and spouse	145(33.3)	90.06 ± 10.20		
Self-reported sleep quality			105.855	<0.001
Worse	96(22.0)	75.10 ± 9.62		
Generally	233(53.4)	84.21 ± 10.75		
Good	107(24.6)	93.75 ± 8.65		
Whether the patient or family members have worked in healthcare			−12.581	<0.001
No	308(70.6)	80.87 ± 11.01		
Yes	128(29.4)	93.40 ± 8.75		
Receipt of chronic disease knowledge guidance			0.939	<0.001
No	158(36.2)	75.29 ± 9.56		
Yes	278(63.8)	89.81 ± 9.61		
Smoking situation			1.487	0.227
Never smoked	202(46.3)	85.60 ± 11.82		
Have given up smoking	122(28.0)	83.71 ± 11.80		
Still smoking	112(25.7)	83.56 ± 11.92		
Drinking situation			1.501	0.224
Never drank	207(47.5)	85.57 ± 11.93		
Have given up drinking	109(25.0)	83.88 ± 11.68		
Still drinking	120(27.5)	83.40 ± 11.83		
Number of chronic diseases			41.461	<0.001
1	138(31.7)	90.18 ± 9.95		
2	161(36.9)	85.26 ± 10.49		
≥3	137(31.4)	78.04 ± 12.04		
Disease duration (years)			75.942	<0.001
<5	202(46.3)	89.81 ± 10.05		
5 ~ <10	105(24.1)	85.35 ± 9.75		
≥10	129(29.6)	75.65 ± 10.84		
Family history of cardiovascular disease			4.296	<0.001
No	121(27.8)	88.17 ± 10.40		
Yes	315(72.2)	83.16 ± 12.10		
Previous hospitalizations for coronary heart disease			54.819	<0.001
0	100(22.9)	91.26 ± 9.40		
1 ~ 2	262(60.1)	84.89 ± 10.91		
≥3	74(17.0)	74.27 ± 10.44		
Number of interventional treatments			44.652	<0.001
1	268(61.5)	88.34 ± 10.61		
2	142(32.5)	79.23 ± 11.25		
≥3	26(6.0)	74.58 ± 10.50		
Number of stent implants			41.993	<0.001
1	129(29.6)	90.07 ± 10.50		
2	143(32.8)	86.50 ± 10.06		
≥3	164(37.6)	78.50 ± 11.66		

To assess the representativeness of our study sample for the Chinese PCI post-procedure population, we compared its key demographic and clinical characteristics with data from an authoritative national PCI registry study. The mean age of patients in this study was (65.77 ± 9.20) years, with males accounting for (58.9%). These figures closely align with the reported mean age (approximately 63.3 years) and a higher male proportion among Chinese PCI patients (34). Therefore, the included sample demonstrates good comparability with the broader Chinese post-PCI population in terms of primary demographic characteristics.

### 3.2 PCI postoperative patient health promotion behaviors, health literacy, psychological resilience, and perceived social support scores

The health promotion behavior score for patients after PCI was (84.55 ± 11.86) points, the health literacy score was (71.48 ± 10.37) points, the psychological resilience score was (25.44 ± 4.11) points, and the perceived social support score was (48.71 ± 4.91) (see Table 2).

**Table 2:** Scores for health promotion behaviors, health literacy, psychological resilience, and perceived social support among patients after PCI.

Items	Number of entries	Scoring range	Score	Entries evenly distributed
Health promotion behaviors	40	40 ~ 160	84.55 ± 11.86	2.11 ± 0.30
Nutrition	6	6 ~ 24	15.31 ± 1.73	2.55 ± 0.29
Physical activity	8	8 ~ 32	15.73 ± 3.20	1.97 ± 0.40
Health responsibility	11	11 ~ 44	22.76 ± 3.51	2.07 ± 0.32
Interpersonal relationships	5	5 ~ 20	11.03 ± 1.99	2.21 ± 0.40
Spiritual growth	5	5 ~ 20	8.82 ± 2.18	1.76 ± 0.44
Stress management	5	5 ~ 20	10.90 ± 1.60	2.18 ± 0.32
Health literacy	24	24 ~ 120	71.48 ± 10.37	2.98 ± 0.43
Psychological resilience	10	0 ~ 40	25.44 ± 4.11	2.54 ± 0.41
Perceived social support	12	12 ~ 84	48.71 ± 4.91	4.06 ± 0.41

### 3.3 Univariate analysis of health promotion behaviors among patients following PCI

Univariate analysis results indicate that factors such as age, BMI, marital status, educational level, occupational status, place of residence, medical payment methods, average monthly family income, housing conditions, self-reported sleep quality, whether the patient or family members have worked in healthcare, receipt of chronic disease knowledge guidance, number of chronic conditions, disease duration, family history of cardiovascular disease, previous hospitalizations for coronary heart disease, number of interventional treatments, number of stent implants. These differences were statistically significant (*p* < 0.05), as shown in Table 1.

### 3.4 Correlation analysis of health literacy, psychological resilience, perceived social support, and health promotion behaviors among patients after PCI surgery

Pearson correlation analysis revealed that health promotion behaviors among patients after PCI were positively correlated with health literacy (*r* = 0.809, *p* < 0.01), psychological resilience (*r* = 0.801, *p* < 0.01), and perceived social support (*r* = 0.747, *p* < 0.01) (see Table 3).

**Table 3:** Correlation between health literacy, psychological resilience, perceived social support, and health promotion behaviors in patients after PCI (*n* = 436, *r*).

Items	Health promotion behaviors	Health literacy	Psychological resilience	Perceived social support
Health promotion behaviors	1			
Health literacy	0.809**	1		
Psychological resilience	0.801**	0.843**	1	
Perceived social support	0.747**	0.794**	0.794**	1

**P < 0.01.

### 3.5 Multivariate stepwise linear regression analysis of health promotion behaviors in patients after PCI surgery

Using health promotion behavior scores as the dependent variable, statistically significant variables from univariate and correlation analyses were incorporated as independent variables into a multiple stepwise linear regression model (*α*_in_ = 0.05, *α*_out_ = 0.10). Hypothesis testing for the regression model was first conducted: the Durbin-Watson statistic value approached 2, indicating residual independence. The multicollinearity analysis revealed variance inflation factors ranging from 1.305 to 4.532, all below 5, confirming the absence of multicollinearity among independent variables. The scatterplot of standardized residuals versus standardized predicted values showed residuals randomly and uniformly distributed on both sides of the zero line, indicating compliance with the assumption of homoscedasticity.

The results of the multiple stepwise linear regression analysis indicate that, based on health ecology theory, within the individual traits layer, age and health literacy are factors influencing health promotion behaviors among PCI patients post-surgery. Within the psychological and behavioral characteristics layer, self-rated sleep quality and psychological resilience are factors influencing health promotion behaviors among PCI patients post-surgery. At the interpersonal network layer, receiving chronic disease knowledge guidance and perceived social support were influencing factors for health promotion behaviors among PCI patients. At the living and working conditions layer, occupational status and whether the patient or family members have worked in healthcare were influencing factors for health promotion behaviors among PCI patients (*p* < 0.05). These factors collectively explained 77.6% of the total variance, as shown in Table 4.

**Table 4:** Multivariate stepwise linear regression analysis of health promotion behaviors in patients after PCI (*n* = 436).

Variables	*B*	SE	*β*	95%CI	*t*	*P*
Constant	11.341	3.439	–	(4.581, 18.101)	3.297	0.001
Age	−1.151	0.417	−0.081	(−1.972, −0.331)	−2.758	0.006
Occupational status	1.667	0.809	0.059	(0.077, 3.257)	2.061	0.040
Self-rated sleep quality	2.326	0.458	0.134	(1.426, 3.226)	5.078	<0.001
Whether the patient or family members have worked in healthcare	2.411	0.674	0.088	(0.955, 3.604)	3.382	0.001
Receipt of chronic disease knowledge guidance	4.687	0.669	0.190	(3.372, 6.001)	7.010	<0.001
Health literacy	0.328	0.055	0.287	(0.220, 0.437)	5.945	<0.001
Psychological resilience	0.751	0.131	0.260	(0.494, 1.008)	5.744	<0.001
Perceived social support	0.303	0.095	0.125	(0.116, 0.489)	3.190	0.002

*R*^2^ = 0.780, adjusted *R*^2^ = 0.776, *F* = 189.480, *p* < 0.001.

## 4 Discussion

### 4.1 Patients’ health promotion behaviors following PCI surgery are at a moderate level

The results of this study indicate that the health promotion behavior scores of patients after PCI were (84.55 ± 11.86) points, with an average score per item of (2.11 ± 0.30) points, reflecting a moderate level. However, this score remains lower than the findings reported by Gong et al. (35) in their survey of patients with coronary heart disease (91.30 ± 11.83). Analysis reveals two key factors. First, 68.6% of PCI patients in this study were aged 60 years or older. With advancing age, most older adult patients experience a gradual decline in physical function and cognitive abilities, potentially leading to greater difficulties in executing health behaviors and, consequently, affecting their motivation to engage in health promotion activities (36). On the other hand, most PCI patients in this study had lower educational attainment, which may have limited their ability to access, comprehend, and utilize health information, thereby hindering their mastery of scientific health management methods (37). In contrast, Gong et al.’s study showed that patients predominantly in the middle-aged and young age groups with educational levels at or above high school/vocational school demonstrated stronger health awareness, information acquisition capabilities, and self-management skills (35). Consequently, they were better able to establish healthy lifestyles, resulting in higher levels of health-promoting behaviors. Furthermore, the findings of this study also fall below those of relevant international research. For instance, a study of patients undergoing PCI in European countries revealed higher levels of health-promoting behaviors, which may be closely associated with their highly systematized cardiac rehabilitation referral processes, nationwide health literacy education programs, and extensive social support networks (13). Among the six dimensions of health promotion behaviors, the nutrition dimension scored the highest average (2.55 ± 0.29 points). This may be attributed to increased public health awareness driven by economic development and rising living standards, with a growing recognition of the importance of nutrition in maintaining physical health, enabling proactive adherence to healthy dietary principles for disease management. Additionally, the average score for the spiritual growth dimension was the lowest at (1.76 ± 0.44). This may be attributed to the fact that most PCI postoperative patients in this study were older and had lower educational attainment. Such patients often prioritize physical recovery while overlooking the importance of spiritual growth and psychological adjustment for overall health. Secondly, due to limitations in educational attainment and cognitive abilities, patients may lack access to and utilization of mental health-related knowledge, making it difficult for them to proactively address their own psychological needs, resulting in lower scores on the spiritual growth dimension (9).

Therefore, healthcare providers should prioritize comprehensive guidance on health promotion behaviors for patients following PCI procedures. Tailored health promotion plans should be developed based on individual differences such as age, educational background, lifestyle habits, and disease type. For instance, simplify language and steps for older patients with lower educational attainment, using illustrated, easy-to-understand methods to explain health knowledge, ensuring patients can comprehend and implement the guidance. Second, health education content should be enriched. Beyond conventional topics such as diet, exercise, and medication, advice on mental well-being should also be incorporated. This includes offering relaxation techniques, meditation, and mindfulness practices to help patients alleviate psychological stress. Such a multidimensional approach promotes health and comprehensively improves patients’ health-promoting behaviors.

### 4.2 Multiple factors influence post-PCI patient health promotion behaviors

#### 4.2.1 Individual traits layer

In health ecology theory, individual characteristics refer to innate traits or relatively stable features formed through long-term life experiences. They constitute the most fundamental proximal determinants of health behaviors, encompassing an individual’s physiological, cognitive, and psychological resources (21). The results of this study indicate that older patients who have undergone PCI exhibit lower levels of health-promoting behaviors, consistent with findings from previous research (36). Analysis suggests that as individuals age, various physiological functions gradually decline, leading to reduced self-care capabilities. Older adult patients also frequently experience cognitive impairments, such as memory loss and attention deficits, which can hinder their ability to comprehend, retain, and apply health information, thereby impeding the effective practice of health behaviors (38). Additionally, older adult patients frequently suffer from multiple chronic conditions requiring concurrent medication regimens. Drug side effects and complex medication schedules may further diminish their capacity to execute health behaviors (39). Consequently, healthcare providers should prioritize older PCI patients, employing plain language and diverse educational approaches to disseminate post-PCI rehabilitation knowledge and precautions, thereby promoting improvements in health-promoting behaviors.

The findings of this study indicate that patients with higher health literacy exhibit greater levels of health-promoting behaviors following PCI, consistent with previous research (40). Health literacy refers to an individual’s ability to access, understand, and apply health information and services to make informed judgments and decisions that promote personal well-being. It constitutes a relatively stable personal capacity or knowledge reserve, falling under the “individual trait layer” dimension within the health ecology theory (41). Patients with higher health literacy can acquire and comprehend health knowledge, develop positive health beliefs and attitudes, gain clearer awareness of their self-management capabilities, and believe they can improve their health status through health-promoting behaviors. Previous studies also indicate that patients with higher health literacy typically have a better understanding of disease-related knowledge, master self-management skills, and are more likely to adopt healthy behaviors actively (42). This not only helps improve disease prognosis but also enhances patients’ overall quality of life. Therefore, healthcare providers should deliver comprehensive health education to patients following PCI procedures. By coordinating efforts across hospitals, homes, and communities, they should disseminate disease-related knowledge to patients, helping them develop scientifically grounded health awareness. Through regular follow-ups and feedback, healthcare providers can reinforce patients’ health beliefs, stimulate their intrinsic motivation for behavioral change, thereby enhancing their health literacy and promoting improvements in health-promoting behaviors.

#### 4.2.2 Psychological and behavioral characteristics layer

In health ecology theory, psychological and behavioral characteristics represent the integrated manifestation of an individual’s stable internal motivations and external expressions within health-related contexts (21). The findings of this study indicate that patients with PCI who self-reported good sleep quality exhibited higher levels of health-promoting behaviors, consistent with previous research (43). Adequate sleep facilitates physical repair and energy restoration, enabling patients to engage in health-promoting activities with greater vigor and stamina. The sleep restoration and repair theory further emphasizes that sleep is a crucial process for physical and mental recovery. High-quality sleep supports the functional recovery of the heart and other organs, reduces inflammatory responses, and enhances immune function (44). Related studies also indicate that good sleep improves cognitive function and decision-making abilities, enabling individuals to understand better and execute health management plans (45). Therefore, healthcare providers should prioritize assessing sleep quality in PCI patients, strengthen sleep health education, and develop personalized sleep intervention plans based on individual circumstances. Methods such as cognitive behavioral therapy or relaxation training can improve sleep quality, thereby promoting the establishment and maintenance of good sleep habits.

The findings of this study indicate that patients with PCI who exhibit higher psychological resilience show greater levels of health-promoting behaviors, consistent with previous research (46). Psychological resilience refers to an individual’s capacity to effectively cope with and adapt to trauma, adversity, threats, or stress (30). As a protective resource, greater psychological resilience enables patients to actively cope with disease-related stress, effectively regulate negative emotions, and mitigate adverse psychological impacts. This fosters a positive mindset and encourages more proactive engagement in health-promoting behaviors (47). Research also indicates that patients with high psychological resilience are more likely to adhere to medical advice regarding rehabilitation exercises, balanced diets, and regular physical activity, thereby improving their physical condition and quality of life (48). Therefore, healthcare providers can offer patients essential psychological support services. Through counseling and cognitive behavioral therapy, they can help patients adjust their perceptions of the disease. Simultaneously, healthcare providers should focus on building trusting relationships with patients. By patiently listening and communicating with empathy, they can understand patients’ psychological needs and life challenges, enhance their sense of self-efficacy and confidence in behavioral change, and improve adherence to health-promoting behaviors.

#### 4.2.3 Interpersonal network layer

In health ecology theory, the interpersonal network layer refers to the social relationship system surrounding an individual that provides informational, emotional, and instrumental support (21). The results of this study indicate that patients with PCI who received guidance on chronic disease knowledge demonstrated higher levels of health-promoting behaviors. Analysis suggests that patients who received such guidance typically possess more comprehensive disease knowledge, often leading to a deeper understanding of the severity of their condition and the importance of health behaviors. This fosters the development of positive health beliefs, which in turn support patients in maintaining health behaviors in daily life, thereby enhancing their health promotion behavior levels (49). Therefore, healthcare providers should strengthen public education on chronic disease knowledge for PCI patients. During hospitalization, tiered education should be provided based on patients’ educational levels. After discharge, establish regular follow-up mechanisms to promote adherence to health management. Additionally, leveraging new media platforms to disseminate accessible health education content, designing interactive modules such as Q&A sessions and health check-ins to encourage patient engagement, and implementing positive feedback mechanisms can facilitate the internalization of health beliefs, ultimately promoting improved health behaviors.

The findings of this study suggest that patients with PCI who perceive higher levels of social support exhibit more health-promoting behaviors, consistent with previous research (50). Perceived social support refers to an individual’s subjective perception of the extent to which support can be obtained from family, friends, and other external sources. It constitutes the “interpersonal network layer” dimension within the health ecology theory (51). The social support buffering theory posits that social support acts as a buffering mechanism, mitigating the negative impact of stress or challenges on individuals and promoting their adaptation and healthy development (52). On the one hand, patients with higher perceived social support are more attuned to the care and support from their social networks, including family, friends, and healthcare providers. This support alleviates psychological burdens, eases anxiety and depression, and fosters a more proactive attitude toward managing their illness (53). On the other hand, patients with high perceived social support are adept at seeking assistance and advice from their support networks when encountering difficulties or setbacks during their illness, which encourages them to adopt positive health-promoting behaviors (54). Therefore, healthcare providers should encourage patients’ families to actively participate in the patient’s rehabilitation plan actively, thereby enhancing family support. Secondly, healthcare providers can establish health management groups for consultation through internet platforms, such as social media and online media. This creates a multi-channel communication platform that connects patients with healthcare providers, family members, and fellow patients, fostering a supportive environment both internally and externally to promote health-promoting behaviors among patients.

#### 4.2.4 Work and living conditions layer

In health ecology theory, the work and living conditions layer refers to the material resources, social roles, and everyday environments that shape individuals’ health opportunities and constraints (21). The findings indicate that employed patients who undergo PCI exhibit higher levels of health-promoting behaviors. Analysis reveals two primary reasons: on the one hand, employed patients typically possess stable income sources, providing them with a stronger material foundation to implement healthy behaviors; on the other hand, employed patients are generally younger, demonstrating a more substantial capacity to absorb and comprehend health knowledge. They also have greater access to health information and broader social support networks, which facilitate more informed health decisions and promote healthier behaviors (55). However, other studies suggest that unemployed patients exhibit higher levels of health-promoting behaviors. This may be attributed to unemployed patients having more time and energy to dedicate to self-health management (36). Therefore, the impact of employment status on health promotion behaviors warrants further validation. This suggests that healthcare providers should fully consider the health needs, behavioral habits, and knowledge levels of patients with different employment statuses. They should develop personalized health promotion plans based on patients’ employment status and individual preferences, encourage patients to undergo regular check-ups and follow-ups, dynamically assess patients’ health conditions, and promptly adjust health promotion plans to ensure the effective implementation of health promotion behaviors.

The results of this study indicate that patients undergoing PCI or their family members with healthcare work experience exhibit higher levels of health promotion behaviors postoperatively. Analysis suggests that patients or family members with healthcare experience typically possess greater medical knowledge and skills, have more channels for accessing health information, and demonstrate a more profound understanding of disease cognition, treatment, and preventive measures. This facilitates better disease monitoring and self-management, encouraging active participation in health-promoting behaviors (9). Thus, adequate and effective health management knowledge is crucial for promoting patients’ health behaviors. This suggests that healthcare providers should establish effective communication channels with patients. Leveraging the internet and mobile health technologies, they can develop relevant health management apps or online platforms to provide patients and their families with convenient access to health information and communication platforms. This ensures patients and their families can effectively acquire health knowledge and management skills, promptly address issues during the recovery process, and create favorable conditions for the patient’s postoperative rehabilitation.

## 5 Limitations

This study also has certain limitations. First, this study exclusively surveyed patients who underwent PCI for coronary heart disease at one tertiary-level general hospital in Sichuan Province, China, resulting in limited sample representativeness. Although, as previously mentioned, the core demographic characteristics of this study’s sample are comparable to national data, the sample originates from a single center in one region. Consequently, the findings may not fully represent PCI patient populations treated at primary-level hospitals or in areas with varying levels of economic development. Future research should conduct large-scale, multicenter investigations to explore variations in health promotion behaviors among PCI patients across different regions, economic levels, and hospital tiers. Secondly, this study is cross-sectional in nature, capable of revealing associations between factors but unable to infer causality. It also cannot assess the longitudinal trajectory of health promotion behaviors among patients following PCI. Future longitudinal studies could further explore the trajectory of health promotion behaviors in post-PCI patients to better guide clinical nursing practice.

## 6 Conclusion

In summary, health promotion behaviors among patients after PCI are at a moderate level and influenced by multiple factors, including age, occupational status, self-rated sleep quality, whether the patient or family members have worked in healthcare, receipt of chronic disease knowledge guidance, health literacy, psychological resilience, and perceived social support. Therefore, it is suggested that comprehensive interventions targeting PCI patients should be implemented from multiple perspectives—individual, family, and societal—tailored to their specific disease characteristics and needs, thereby improving their health promotion behaviors.

## Data Availability

The original contributions presented in the study are included in the article/Supplementary material, further inquiries can be directed to the corresponding authors.
